# Supporting general practitioners in the assessment and management of suicide risk in young people: an evaluation of an educational resource in primary care

**DOI:** 10.1017/S1463423622000433

**Published:** 2022-08-31

**Authors:** Maria Michail, Aimee Cairns, Emma Preece, Faraz Mughal

**Affiliations:** 1 Institute for Mental Health, School of Psychology, University of Birmingham, Birmingham, UK; 2 Faculty of Health and Medicine, Lancaster University, Lancaster, UK; 3 School of Psychology, University of Birmingham, Birmingham, UK; 4 School of Medicine, Keele University, Keele, Staffordshire, UK; 5 Affiliate, NIHR Greater Manchester Patient Safety Translational Research Centre, The University of Manchester, Manchester Academic Health Science Centre, Manchester, UK; 6 Honorary Clinical Research Fellow, Unit of Academic Primary Care, University of Warwick, Coventry, UK

**Keywords:** general practice, medical education, primary care, suicide, youth mental health, young people

## Abstract

**Aim:**

To conduct a local evaluation of the use of the educational resource: Suicide in Children and Young People: Tips for GPs, in practice and its impact on General Practitioners (GPs)’ clinical decision making.

**Background:**

This Royal College of General Practitioners (RCGP) resource was developed to support GPs in the assessment and management of suicide risk in young people.

**Method:**

The dissemination of the educational resource took place over a nine month period (February 2018–October 2018) across two Clinical Commissioning Groups in West Midlands. Subsequently, a survey questionnaire on GPs’ experiences of using the resource was sent to GPs in both Clinical Commissioning Groups (CCGs).

**Findings:**

Sixty-two GPs completed the survey: 21% reported that they had used the resource; most commonly for: (1) information; (2) assessing a young person; and (3) signposting themselves and young people to relevant resources. Five out of thirteen GPs (38.5%), who responded to the question about whether the resource had an impact on their clinical decision making, reported that it did; four (30.7%) responded that it did not; and four (30.7%) did not answer this question. Twenty out of thirty-two GPs (62.5%) agreed that suicide prevention training should be part of their NHS revalidation cycle. The generalizability of the findings is limited by the small sample size and possible response and social desirability bias. The survey questionnaire was not validated. Despite the limitations, this work can be useful in informing a future large-scale evaluation of the RCGP online resource to identify barriers and facilitators to its implementation.

## Introduction

Suicide is a growing public health concern and the fourth leading cause of mortality in young people aged 15–19 globally (WHO, 2021). In the UK, suicide rates among those under 25 have steadily increased over recent years (ONS, 2019). Although mental ill health (e.g., depression) is a key risk factor for suicide, many of the determinants which increase vulnerability to suicide during late adolescence or early adulthood have social roots such as loss of employment, financial difficulties, social isolation, and bullying (HQIP, 2017). This means that suicide intervention and prevention strategies need to be informed by a multiagency public health approach with a strong focus on enhanced and integrated models of primary and community mental health services (NHS England, 2019; Michail *et al.*, 2020).

The role of primary care in identifying and supporting those at-risk of suicide, including young people, has been extensively documented (Michail *et al.*, 2016; 2020; Mughal *et al.*, 2019) and supported by the wider policy context in the UK (DHSC, 2012; NHS England, 2019). Research (Bellairs-Walsh *et al.*, 2020; Farr *et al.*, 2021; Mughal *et al*., 2021) shows that, although young people’s experiences of seeking help from General Practitioners (GP) for self-harm and suicidal behaviour are mixed, GPs remain a valued source for help-seeking. GPs also consider their role vital in supporting young people at-risk of suicide over and above acting as gatekeepers for access to specialist services (Michail *et al.*, 2020). The challenges, however, GPs face in this role are many and among those, lack of specialist training and resources on suicide risk assessment and management have been consistently highlighted as significant barriers to providing optimal care to young people (Centre for Mental Health & Samaritans, 2019; Michail *et al*., 2020).

### Suicide in Children and Young People: Tips for GPs

In response to the aforementioned challenges, the Royal College of General Practitioners (RCGP) commissioned the development of an online educational resource to support GPs in the assessment and management of suicide risk in young people. The *Suicide in Children and Young People: Tips for GPs* is a brief (circa 10 mins to read), practical, and user-friendly resource, in the format of an article, developed by MM, FM, and AM; and informed by youth suicide prevention evidence (Michail & Tait, 2016; Michail *et al*., 2017), with input from RCGP Clinical Advisors. The resource consists of three sections: (i) an introductory section providing GPs with information about well-documented risk factors for suicide in children and young people; (ii) ten suggestions (“top tips”) on how to assess and manage suicide risk in a consultation: how to address confidentiality; asking young people about suicidal ideation; developing a safety plan; and key legal and ethical issues in relation to assessing capacity, information sharing, and safeguarding; and (iii) signposting GPs to key resources for supporting young people at-risk of suicide. This resource was launched by RCGP in 2018 as part of the Mental Health Clinical Priority and is available open access within the RCGP Mental Health Toolkit.

## Aim

The aim of this study was to conduct a local evaluation of the use of the RCGP Suicide in Children and Young People: Tips for GPs (Supplementary Material) resource in practice and its impact on GPs’ clinical decision making. Findings from the local evaluation will inform optimal ways of implementing the educational resource at-scale in routine practice and support a national evaluation.

## Methods

### Setting

The evaluation took place across two Clinical Commissioning Groups (CCGs) in West Midlands: Birmingham and Solihull (BSOL) CCG, the largest clinically led commissioning organisation in England with 164 member general practices; and Sandwell and West Birmingham (SWB) CCG (now called Black Country & West Birmingham CCG) with 84 member general practices.

### Design

Phase 1 of this project involved the dissemination of the educational resource over a nine month period (February 2018–October 2018) to CCG practices using a multimethod approach. This included emailing and posting copies of the resource to all practices; dissemination via CCGs’ newsletters and targeted social media. MM and FM attended CCG teaching afternoon and locality meetings; and FM attended RCGP Midlands Faculty Board meetings. The authors were allocated a slot between 15 and 30 mins and presented the need and rationale behind the design of the educational resource; highlighted the role of primary care in suicide prevention including challenges and opportunities; and how the resource could be used by GPs to facilitate assessment and management of suicide risk among young people. GPs were invited to ask questions during the presentations and reflect on their clinical experience of managing suicidal presentations in primary care.

Phase 2 involved a cross-sectional survey (online or face-to-face) on GPs’ views and experiences of using the resource in practice and its impact on GPs’ clinical decision making.

### Material

A structured, self-administered survey (Supplementary Material) comprising 14 questions was developed by the authors (including two practising GPs) with the aim of examining: how many GPs across the two CCGs used the educational resource since its launch; how often the educational resource was used; reasons for use (such as to support assessment of young people); perceived impact on decision making; and resource satisfaction. The survey was piloted with a convenience sample of five GPs from BSOL CCG (approached through professional networks of the authors) to assess face validity. The last question of the survey asked GPs about their views on whether training in suicide prevention should be a mandatory part of the NHS GP revalidation cycle.

### Data collection

Cluster selective sampling was adopted, whereby clusters were the different CCGs across West Midlands. We randomly picked BSOL and SWB CCG and GPs at the time of February 2018–October 2018 across the two CCGs were invited to participate in the survey via CCG mailing lists, newsletters, and during lunchtime practice meetings. Responses were anonymized and completion took approximately 10 mins. The survey was administered both online using Qualtrics and face to face (e.g., during lunchtime practice meetings) to maximize recruitment opportunities.

### Data analysis

Response data were exported from Qualtrics to the Statistical Package for the Social Sciences (SPSS) version 25. Descriptive statistics including frequencies and percentages are reported. Free-text responses are presented as anonymized quotes.

## Results

### Sample population

The demographic characteristics of the sample are presented in Table 1. A total of 62 GPs completed the survey (35 online and 27 face to face); 25 of whom were males and 29 females. The majority of the GPs identified as White British (45.2%); had 15–20 years of professional experience (24.2%); were from large practices (≥9000 patients) (59.7%); and reported that they had not received any community suicide prevention training in the past 5 years (66.1%).

**Table 1.** Demographic characteristics of sample

	Sample (*n* = 62)
Age	
21–29	2 (3.2%)
30–39	19 (30.6%)
40–49	17 (27.4%)
50–59	13 (21%)
60+	3 (4.8%)
Missing	8 (12.9%)
Gender	
Male	25 (40.3%)
Female	29 (46.8%)
Other	1 (1.6%)
Missing	7 (11.3%)
Professional experience (years since medical qualification)	
<5	3 (4.8%)
5–10	7 (11.3%)
10–15	8 (12.9%)
15–20	15 (24.2%)
20–25	5 (8.1%)
25–30	5 (8.1%)
30–35	10 (16.1%)
35+	2 (3.2%)
Missing	7 (11.3%)
Ethnicity	
White British	28 (45.2%)
White Irish	1 (1.6%)
Other White	2 (3.2%)
White and Asian	1 (1.6%)
Asian/Asian British	1 (1.6%)
Asian/Asian British Indian	12 (19.4%)
Asian/Asian British Pakistani	5 (8.1%)
Other Asian	4 (6.4%)
Other ethnic group	1 (1.6%)
Missing	7 (11.3%)
Practice size^a^	
<3000	0 (0%)
3000–5999	9 (14.5%)
6000–8999	8 (12.9%)
≥9000	37 (59.7%)
Missing	8 (12.9%)
Attendance of any community suicide prevention training in the past 5 years	
Yes	14 (22.6%)
No	41 (66.1%)
Missing	7 (11.3%)

a Sampe size for this variable *n* = 54.

### Use of educational resource in practice and perceived impact on decision making

Twenty-one per cent (21%; 13/62) of the sample confirmed that they had used the RCGP resource “Suicide in Children and Young People: Tips for GPs” in practice since launch. Of those, 46.1% (*n* = 6) used the resource for information; 15.4% (*n* = 2) for assessing a young person; and 7.7% for signposting themselves (*n* = 1) and young people to relevant resources (*n* = 1) (Table 2). Five out of thirteen GPs (38.5%), who responded to the question about whether the resource had an impact on their clinical decision making, reported that using the educational resource in a consultation with a young person informed their clinical practice. One GP said (free text response) “I felt more confident in assessing suicide risk and was able to signpost patients to online resources.” Another GP described that it “Helped with better communication with a young person and structure of assessment.” Among those who reported that the resource had an impact on a clinical decision, 30.7% (4/13) rated it as being somewhat helpful and 46.1% (6/13) as extremely helpful.

**Table 2.** Reasons for using the educational resource

	Sample (*n* = 13)
For information only	6 (46.1%)
To be used in the assessment of a young person	2 (15.4%)
To be used in the shared management of a young person	0 (0%)
For signposting to resources mentioned for young person	1 (7.7%)
To refer to resources mentioned for myself	1 (7.7%)
Missing	3 (23.1%)

The majority of GPs (67.7%; 42/62) who completed the survey had not used the educational resource. The main reason for this was not being familiar with or aware of the resource (54.8%; 23/42). Free text responses (Supplementary Material) also indicated time constraints as a common reason for not being able to use the resource in consultations with young people. The last question of the survey asked GPs to indicate whether they thought training in suicide prevention should be a mandatory part of the NHS validation cycle for GPs. Of those GPs who answered this question (*n* = 32), 62.5% responded “yes”; 12.5% “no”; and 25% were not sure.

## Discussion

This small scale regional evaluation sought to examine the use of a RCGP online educational resource to support GPs in the assessment and management of suicide risk in consultations with young people; and its impact on clinical decision-making.

The majority of GPs who took part in the survey had not attended or undertaken any suicide prevention training in the last five years. Suicide is a rare event; however, there has been an increased frequency of self-harm presentations among young people in primary care; and young people who self-harm present to their GP the most in the NHS (Marchant *et al*., 2020). Although the effectiveness of suicide prevention training programs for GPs remains unclear (Milner *et al*., 2017), GPs have consistently identified the need for further education and training that is easy to access, feasible, and grounded in the realities of general practice (Mughal *et al.*, 2020; Samaritans, 2020). The findings of this evaluation reinforce this need: the majority of GPs agreed suicide prevention training should be a mandatory part of their appraisal and revalidation cycle. Commissioned evidence-informed suicide prevention training for GPs, adopting a hybrid model, would enable GPs to meet such an appraisal requirement.

The most common reasons for using the educational resource in practice were (i) for information; (ii) for assessing suicide risk in young people and (iii) for signposting. The feedback by those GPs was positive citing two ways in which the resource supported their decision making: (i) by increasing their confidence in assessing suicide risk in young people and (ii) facilitating better communication with young people. The ability to communicate effectively with children and young people across different ages and development stages is a core professional competency relevant to health and social care professionals working with young people who self-harm and/or have suicidal experiences (NHS England, 2018).

Verbal and nonverbal communication skills including asking young people sensitive questions, active listening, showing empathy and compassion are among those elements perceived by young people and GPs to facilitate rapport and engagement; but also areas where further training and support are warranted (Bellairs-Walsh *et al.*, 2020; Michail *et al*., 2020; Farr *et al*., 2021).

Our findings show promise for the acceptability and usefulness of this RCGP education resource in clinical practice. It is worth noting, however, that the majority of GPs who completed the survey were not aware of or familiar with the resource. This opens up opportunities for strategic and targeted dissemination. The appointment of GP champions (or knowledge brokers), for example, could significantly facilitate the promotion and uptake of the RCGP resource in clinical practice by (i) leading on both written and verbal dissemination to a clinician audience through local and national primary care forums and publications in peer-reviewed and professional journals; (ii) being in strategic positions to influence service commissioning and delivery which will facilitate resource implementation; and (iii) acting as role models for peers by adopting the resource in their usual practice.

### Limitations

This study reflects a regional evaluation, and the generalizability of the findings is restricted by the small sample size. The survey questionnaire was designed specifically for the purposes of this evaluation and construct validity was not assessed. The survey was piloted with a convenience sample of GPs approached through the authors’ professional networks and therefore selection and social desirability bias is possible. We are unable to provide an accurate response rate as it is not possible to know whether our survey invitation reached all GPs across the two CCGs. It is also plausible that from those GPs who received the invite only those with a particular interest in the topic completed the survey.

Despite its limitations, this work can be useful in informing a future large-scale evaluation of this RCGP online resource to identify barriers and facilitators to its implementation.

### Implications for research and practice

A future larger scale evaluation of the resource would need to carefully consider the above limitations particularly the small sample size which reflects previously documented challenges in engaging busy clinicians in research (Pit *et al.*, 2014). The appointment of GP champions (or knowledge brokers), mentioned above, could significantly facilitate the uptake of the RCGP resource in clinical practice.

A larger evaluation of the RCGP online resource could be significantly enhanced by conducting interviews or focus groups with GPs to explore their views and experiences of using the educational resource in consultations with young people; barriers and facilitators of its use; and pathways to implementation in routine practice. Interviews with service commissioners at a national level (e.g., Public Health England and NHS England and Improvement) could provide useful insight into ways of facilitating implementation into routine practice; maximizing opportunities for knowledge mobilization and exchange with Integrated Care Systems (ICSs) nationally.

## Conclusion

This study highlights the need for continued education in suicide risk assessment and management of young people in primary care and offers preliminary findings for the acceptability and clinical utility of the RCGP Suicide in Children and Young People: Tips for GPs resource. Findings from this evaluation can inform optimizing the implementation of this educational resource at-scale in routine practice in the National Health Service (NHS).

## Supplementary material

For supplementary material accompanying this paper visit http://doi.org/10.1017/S1463423622000433. click here to view supplementary material
