# Supplementary material for: Supporting general practitioners in the assessment and management of suicide risk in young people: an evaluation of an educational resource in primary care
Source: Prim Health Care Res Dev. 2022 Aug 31;23:e50. doi: 10.1017/S1463423622000433 (PMC9433951; doi:10.1017/S1463423622000433)
Supplement: Supplementary file 1 [file phcsup.zip › S1463423622000433sup001.pdf]

Royal College of General Practitioners

Suicide in children and young people: Tips for GPs

Dr Maria Michail, Senior Research Fellow in Youth Mental Health, School of Psychology, University of Birmingham.

Dr Faraz Mughal GP, RCGP Clinical Fellow in Mental Health, and NIHR In-Practice Fellow, Keele University.

Background

Suicide is the second leading cause of death among children and young people (CYP) aged 15-29 worldwide (1). In the United Kingdom (UK), suicide accounts for 14% of deaths in 10-19 year olds and 21% of deaths in 20-34 year olds (2). CYP who present to their General Practitioner (GP) are twice as likely to have a mental health problem (3) and thus considering mental illness and suicidal risk is important for all GPs.

Risk factors for suicide in CYP (4, 5):

- Self-harm (over half of CYP who die by suicide have a history of self-harm)
- Previous suicide attempt
- Diagnosis of mental illness most commonly depression or bipolar disorder
- Family discord (parental separation, divorce, domestic violence)
- History of mental illness in family
- Bereavement (especially bereavement by suicide)
- Sexual and gender identity (e.g. lesbian, gay, bisexual, transsexual, transgender, queer)
- History of neglect and abuse including physical, sexual, or emotional
- Looked after/being in care
- Bullying
- Academic pressures, especially related to exams
- Substance abuse

It can be difficult to undertake a comprehensive mental health assessment of a young person in the context of the competing demands of general practice (6). This document offers some suggestions specifically about how to assess and manage suicide risk in a consultation, acknowledging that more detailed mental health assessment may need to take place over multiple consultations and with input from other services.

Top tips for GPS:

1. Know the Young Person

GPs have the advantage of often knowing about a young person's family and social circumstances before they come into the consultation. Focussing on 'at risk' groups such as those listed above can prioritise those most at risk. However, it is important to remember that CYP may experience suicidal ideation even if they are not in a risk group.

2. Communicate Effectively

As all patients, CYP are most likely to express their feelings if they are treated with respect, listened to, and taken seriously in a compassionate, non-judgmental, non-stigmatising approach using age-appropriate language.

3. Explain Confidentiality (and its limits) and Develop Trust

Fear about others finding out is the most common reason that young people withhold information from their GP. An explicit assurance of confidentiality early in the consultation will help this. However, in the context of assessing suicide risk it is important to acknowledge the circumstances under which you may have to share information with others – whilst stating that you would always want to do this with the CYPs agreement, if possible. Developing trust is one of the most important part of the process.

4. Explore the Background

HEADSS (Home, Education, Activities/Employment, Drugs, Suicide, Sex, Safety) (7) provides a useful format for carrying out a brief psychosocial assessment of the main areas relevant to a CYPs life (8). These factors will be important both in evaluating risk but also in identifying support.

5. Assess Mental State

Does the CYP have any current mental health problems (e.g. depression, ADHD, anxiety, substance misuse, eating disorders)? Ask about mood, sleep, screens and appetite.

6. Ask About Suicidal Ideation Directly

In the context of asking about mental state, use open non-judgemental permissive questions to enquire about suicidal ideation (e.g. "I know that it can be difficult to talk about this, but when young people feel down they often think about hurting themselves or even taking their own lives: I wonder if you have had thoughts like that?"). There is no evidence that talking about suicide or self-harm increases the risk (9).

7. Explore Suicidal Ideation more thoroughly

Are these just thoughts or have they specific plans or taken any action?

- Explore feelings of hopelessness (e.g. How do you see the future?)
- Explore any wishes to be dead (e.g. Do you wish you were better off dead?)
- Enquire about suicidal ideas (e.g. Do you have thoughts of ending your life; Can you tell me what you have been thinking?)
- Enquire about suicidal plans (e.g. Have you made plans to end your life?)

- Enquire about suicide-related internet use (e.g. searching for suicide methods; using social media to post messages with suicidal content)
- Explore possible self-harm (e.g. cutting, hitting, pinching, burning, overdosing)
- Explore access to lethal means (e.g. medication, sharp objects)

8. Evaluate the Risk

Balance the likelihood of harm with the protective factors (e.g. family support, friends etc) taking into account other risk factors. This is the most difficult step but equally something that GPs do all the time in other areas of work.

9. Develop a Safety Plan

Young people need to be at the centre of decisions made about their care. Think about co-producing a safety plan over time with the CYP and family (if appropriate). If there is risk, explore the following with the CYP:

- What would keep them safe?
- Who would they go to for help in case of a crisis (parents, siblings, friend)?
- What are their coping strategies?
- Is there need to remove access to means of suicide (e.g. medication)?
- Who else needs to be informed?
- What follow-up will you provide? This is very important if you are going to continue to support the CYP.
- What other services need to be involved and how quickly?

Depending on the assessment of risk and needs, you may want to seek advice and support from your local CAMHS team.

10. Consider Legal and Ethical Issues

Capacity: GPs should assess mental capacity of the CYP. If they feel the CYP does not have capacity they should seek parental input if under age of 16 and further advice about best interests if aged over 16 years and parental input not available.

Information sharing: In line with the Consensus statement (10, 11), GPs should ask young people how they wish information to be shared, and with whom including what should happen if there is concern about suicide risk. When dealing with a young person at imminent risk who might lack capacity to make a decision whether to share information about their suicide risk, GPs should use their professional judgement to determine what it is in the young person’s best interest.

Confidentiality: This needs to be emphasised to all CYP and should be done with the CYP individually. Asking family members or friends to vacate the room for a short time should be undertaken and not seen as a barrier to this.

Safeguarding: If there are concerns over abuse for the CYP and this is identified as a trigger for suicidal risk, GPs must act immediately to liaise with local safeguarding services to

ensure the CYP is safe from future potential harm. A safeguarding and mental health assessment can be undertaken side by side.

Resources for GPs

http://iapdeathsincustody.independent.gov.uk/wp-content/uploads/2015/08/Information-sharing-and-suicide-prevention-–-Consensus-statement.pdf

https://www.psych.ox.ac.uk/research/csr AND http://cebmh.warne.ox.ac.uk/csr/clinicalguide/index.html

https://www.mentalhealth.org.uk/sites/default/files/young_people_mh_problems1.pdf

http://www.connectingwithpeople.org/healthcare-front

https://www.cwmt.org.uk Charlie Waller Memorial Trust

https://www.mentalhealth.org.uk/sites/default/files/young_people_mh_problems1.pdf

http://www.cpft.nhs.uk/U%20R%20CYP%20at%20risk%20of%20selfharm%20and%20suicide%202014%20v1%20electronic.pdf

Useful resources for CYP

Young Minds – Suicidal feelings

https://youngminds.org.uk/find-help/feelings-and-symptoms/suicidal-feelings/

PAPYRUS – Prevention of young suicide

https://www.papyrus-uk.org/

http://www.themix.org.uk/mental-health/self-harm

Samaritans

https://www.samaritans.org/education/young-people

Acknowledgements:

We would like to thank the RCGP Clinical Advisors who provided valuable peer-review.

References

1. World Health Organisation (WHO). Preventing suicide: a global imperative. Geneva: World Health Organisation, 2014.
2. Office for National Statistics (ONS) Deaths registered in England and Wales: 2015. Statistical Bulletin 2016: 1-8.
3. Mughal F, England E. The mental health of young people. British Journal of General Practice, 66 (651).
4. Hawton, K, Saunders, KE, O'Connor, RC. Self-harm and suicide in adolescents. Lancet 2012; 379(9834):237383
5. Suicide by children and young people. National Confidential Inquiry into Suicide and Homicide by People with Mental Illness (NCISH). Manchester: University of Manchester, 2017.

6. Michail M., Tait L. (2016). Exploring general practitioners' views and experiences on suicide risk assessment and management of young people in primary care: a qualitative study in the UK. BMJOpen, 6 (1)

7. Goldenring JM, Cohen E: Getting into adolescent heads. Contemporary Pediatrics, 1988; 5(7):75.

8. National Institute for Health and Clinical Excellence. Self-harm: longer-term management. (Clinical guideline CG133). 2012. http://guidance.nice.org.uk/CG133

9. Dazzi T., Gribble R., Wessely S., Fear NT. (2014). Does asking about suicide and related behaviours induce suicidal ideation? What is evidence? Psychological Medicine, 44 (16), 3361-3

10. Information sharing and suicide prevention. Consensus statement. January 2014. http://iapdeathsincustody.independent.gov.uk/wp-content/uploads/2015/08/Information-sharing-and-suicide-prevention-—-Consensus-statement.pdf

11. Preventing suicide in England: Third progress report of the cross-government outcomes strategy to save lives. DH 2017 https://www.gov.uk/government/uploads/system/uploads/attachment_data/file/582117/Suicide_report_2016_A.pdf
