# Supplementary material for: Supporting general practitioners in the assessment and management of suicide risk in young people: an evaluation of an educational resource in primary care
Source: Prim Health Care Res Dev. 2022 Aug 31;23:e50. doi: 10.1017/S1463423622000433 (PMC9433951; doi:10.1017/S1463423622000433)
Supplement: Supplementary file 1 [file phcsup.zip › S1463423622000433sup002.docx]

**Free-text responses**

|  |  |
| --- | --- |
| **Q8. Other (please specify)** | *Time* |
|  | *Time constraints* |
|  | *Not had opportunity* |
|  | *Time resources* |
|  | *Not done yet, intend to very soon* |
|  | *No real clinical opportunity* |
|  | *Lack of time* |
|  | *Not had yet the opportunity to apply it.* |
|  | *It hasn’t come up in a consultation* |
| **Q12. How did it impact on your clinical decision?** | *Seating in holistic assessment* |
|  | *I felt more confident in assessing suicide risk and was able to signpost patient to online resources.* |
|  | *More confident* |
|  | *Helped me to think about role of education/teacher etc.* |
|  | *Helped with better communication with young person and structure of assessment.* |
