# Supplementary material for: Supporting general practitioners in the assessment and management of suicide risk in young people: an evaluation of an educational resource in primary care
Source: Prim Health Care Res Dev. 2022 Aug 31;23:e50. doi: 10.1017/S1463423622000433 (PMC9433951; doi:10.1017/S1463423622000433)
Supplement: Supplementary file 1 [file phcsup.zip › S1463423622000433sup003.docx]

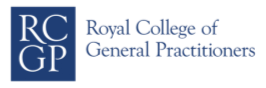

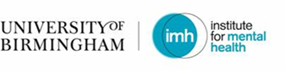


**Suicide in Children and Young People: Tips for GPs**

The Royal College of General Practitioners (RCGP) has recently launched an online educational resource titled "Suicide in Children and Young People: Tips for GPs". This can be accessed here <http://www.rcgp.org.uk/clinical-and-research/about/clinical-news/2018/february/mental-health-in-young-people-top-tips-for-gps.aspx>

**The purpose of this short survey is to seek your views and experiences of using this online resource in consultations with children and young people.**

This evaluation is conducted and led by Dr Maria Michail, University of Birmingham, in collaboration with, Dr Faraz Mughal, NIHR In-Practice Fellow, Primary Care & Health Sciences, Keele University, RCGP Clinical Fellow in Mental Health, and Dr Elizabeth England, GP Mental Health Clinical Lead for SWB CCG & RCGP Mental Health Clinical and Commissioning Lead.

1. **What is your age?**

21-29 _____

30-39 _____

40-49 _____

50-59 _____

60 or older _____

1. **What is your gender?**

Female _____

Male _____

Other _____

1. **Which race/ethnicity best describes you? (Please choose only one)**

White British ___

White Irish ___

White Gypsy or Irish Traveller ___

Other White ___

Mixed/Multiple Ethnic Group ___

White and Black Caribbean ___

White and Black African ___

White and Asian ___

Other Mixed ___

Asian/Asian British ___

Asian/Asian British Indian ___

Asian/Asian British Pakistani ___

Asian/Asian British Bangladeshi ___

Asian/Asian British Chinese ___

Other Asian ___

Black/African/Caribbean/Black British ___

African ___

Caribbean ___

Other Black ___

Other ethnic group ___

Arab ___

Any other ethnic group ___

1. **Professional experience (years since medical qualification)**

<5 ___

5-10 ___

10-15 ___

15-20 ___

20-25 ___

25-30 ___

30-35 ___

35+ ___

1. **What is your practice size?**

< 3000 ___

3000 – 5999 ___

6000 - 8999 ___

> 9000 ___

1. **Have you attended any community suicide prevention training in the last 5 years?**

Yes ___

No ___

1. **Since its publication, have you used the RCGP online resource "*Suicide in Children and Young People: Tips for GPs*" in a consultation with a young person?**

Yes ___

No ___

1. **If No, please let us know why.**

Not a member of RCGP

Not aware of the resource

Not in a helpful format

Other (please specify) ________________________________________________________________

1. **If YES, how often have you used this resource?**

Once ___

Twice ___

3-4 ___

5-6 ___

7-8 ___

9-10 ___

11+ ___

Never ___

1. **Why did you use this resource?**

For information only ____

To be used in this assessment of a young person ____

To be used in the shared management of a young person ____

For signposting to resources mentioned for young person ____

To refer to resources mentioned for myself ____

I have yet to use the educational resource ____

1. **Did it impact on a clinical decision?**

Yes ___

No ___

Not applicable ___

1. **How did it impact on your clinical decision? Please specify below.**
2. **How helpful did you find the online resource?**

Extremely helpful ___

Somewhat helpful ___

Neither helpful/unhelpful ___

Somewhat unhelpful ___

Not helpful at all ___

Not applicable ___

1. **Do you think training in suicide prevention should be a mandatory part of a GP NHS revalidation cycle?**

Yes ___

No ___

Not sure ___

**Thank you for completing this survey.**
